# Organic fertilizer application rates affect rhizosphere microbial communities and yield optimization in potato (*Solanum tuberosum* L. V7)

**DOI:** 10.3389/fmicb.2025.1651178

**Published:** 2025-08-07

**Authors:** Xiaodong Han, Jing Yang, Qi Li, Lan Zhang, Yuankai Li, Yaoyao Gai, Yuying Sang, Ziyi Zhang

**Affiliations:** ^1^College of Life Sciences, Inner Mongolia Agriculture University, Hohhot, China; ^2^Vocational and Technical College of Inner Mongolia Agricultural University, Baotou, China; ^3^Agricultural and Animal Husbandry Technology Extension Center, Chifeng, China

**Keywords:** organic fertilizer, rhizosphere microbiome, potato yield optimization, microbial diversity, sustainable agriculture

## Abstract

**Background:**

Organic fertilizers enhance sustainable agriculture by providing nutrients and supporting microbial communities. However, optimal application rates that maximize potato yield while maintaining rhizosphere microbial diversity remain poorly understood.

**Methods:**

Four organic fertilizer levels (0, 40, 60, and 80% nitrogen replacement) were tested on potato rhizosphere bacterial and fungal communities across three growth stages using high-throughput 16S rDNA and ITS sequencing.

**Results:**

Bacterial richness increased progressively with organic fertilizer rates (80% > 60% > 40% > 0), with principal coordinate analysis revealing distinct community separations and largest differentiation during tuber expansion under 80% treatment. Bacterial and fungal communities were dominated by *Proteobacteria*, *Acidobacteriota*, and *Gemmatimonadota*, and *Ascomycota*, *Mortierellomycota*, and *Basidiomycota,* respectively. T60 maintained optimal balance of beneficial rhizospheric microorganisms and delivers superior yield outcomes compared with other fertilization regime. Potato yield responded quadratically to organic fertilizer application, with optimal yield of 81,020 kg/ha at 51.25% organic fertilizer rate, while bacterial and fungal diversity correlated with yield.

**Conclusion:**

Moderate organic fertilization (50–60% nitrogen replacement) optimizes both rhizosphere microbial diversity and potato productivity through enhanced nutrient cycling efficiency, providing a sustainable approach for potato production systems.

## Graphical abstract

**Figure:**
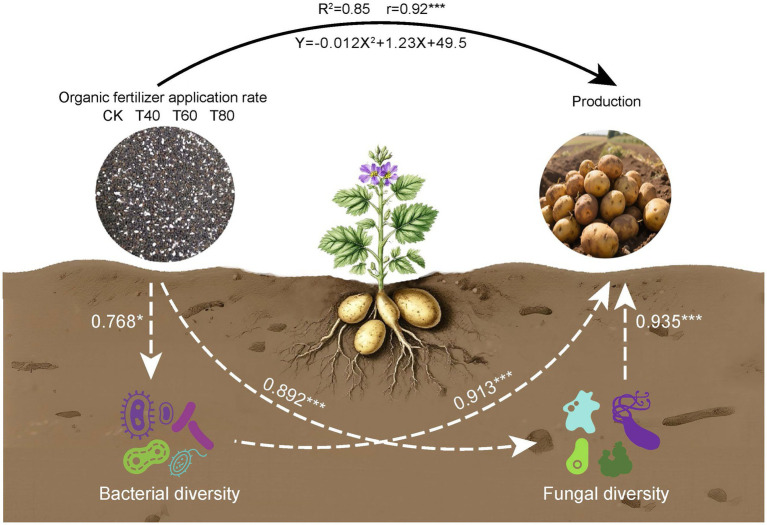
Illustration showing the impact of organic fertilizer on potato plant growth and soil biodiversity.

## 1 Introduction

Organic fertilizers are essential for sustainable agriculture, providing nutrients, improving soil structure, and supporting beneficial microorganisms (Bing et al., 2022; Dong et al., 2022; Cheng et al., 2024). These fertilizers contain essential elements including carbon, nitrogen, and phosphorus, with some types enriched in bioactive substances like humic acids and enzymes (Tang et al., 2012). For instance, humic acid application to Diyala Black fig seedlings enhanced growth and root development while improving soil structure and nutrient availability (Shafaqat and Al-Dolaimi, 2023). A meta-analysis based on 220 studies demonstrated that organic fertilizers promote stable soil aggregation, correlating with increased soil organic carbon and microbial biomass (Liu et al., 2023; Raymond et al., 2023). However, organic fertilizer effectiveness depends heavily on application rates. Optimal amounts enhance crop productivity through steady nutrient release and improved stress tolerance (Ika et al., 2024; Margarida et al., 2022), while excessive application causes soil chemical imbalances, heavy metal accumulation, and harmful microbial growth (Theocharis et al., 2021).

Root-associated microbial communities form the essential biological interface between plants and soil. These diverse assemblages include nitrogen-fixing bacteria, phosphate-solubilizing microorganisms (González-Mancilla et al., 2024), and arbuscular mycorrhizal fungi that provide critical services through nutrient mobilization, plant hormone production, and disease control (Delai et al., 2023; Hamed et al., 2021). Microbial communities decompose complex organic compounds through enzymatic processes, facilitating plant nutrient acquisition via specialized metabolic pathways (Yuanyuan et al., 2023; Hong et al., 2023). The rhizosphere environment responds dynamically to organic fertilizer inputs through altered carbon availability (Bin et al., 2024), pH balance (Shoubiao et al., 2024), and oxygen levels (Zhida et al., 2022), selectively promoting beneficial microbial groups while suppressing pathogens and reshaping interaction networks (An-Hui et al., 2021; Noemí et al., 2022). Despite documented relationships between organic fertilizer application rates and microbial community structure, understanding of specific threshold amounts and connections between microbial functional diversity and crop productivity remains incomplete (Yijie et al., 2023).

Potato (*Solanum tuberosum* L.), the fourth most important staple crop globally, exhibits high dependence on rhizosphere microbes due to its limited root structure and intensive nutritional demands during tuber formation (Feng and Alchanatis, 2016; Martins et al., 2024). Current research has primarily focused on individual organic fertilizer types effects on potato yields while neglecting detailed studies of rhizosphere microbial community responses to varying fertilizer concentrations. Most studies provide only qualitative descriptions of fertilizer concentrations effects on rhizosphere microbial diversity, lacking quantitative analyses from systematic gradient experiments. Researchers have insufficiently characterized how plant growth-promoting rhizobacteria and pathogen-suppressive microbial communities respond to fertilizer gradients (Qiuyun et al., 2024). Additionally, mechanistic connections between microbial community structural changes and potato agricultural performance remain inadequately established through comprehensive frameworks spanning taxonomic composition to metabolic functionality (Jeanne et al., 2019).

This investigation addresses critical knowledge gaps through systematic field experiments examining four organic fertilizer application levels (0, 40, 60, and 80% of total nitrogen input) on potato rhizosphere bacterial and fungal community dynamics using high-throughput 16S rDNA and ITS amplicon sequencing. Our research objectives include measuring the dose-dependent relationships between organic fertilizer application rates and rhizosphere microbial community structure and *α*/*β*-diversity metrics, identifying key microbial species and their response thresholds to organic fertilizer concentration gradients, and developing quantitative models linking microbial community indicators to potato yield optimization. This research tries to examine three fundamental questions whether threshold effects control relationships between organic fertilizer application and microbial diversity, how functional microbial groups respond quantitatively to organic inputs, and the mechanisms by which microbial community changes influence potato yield formation.

## 2 Materials and methods

### 2.1 Experimental site characterization

The field trial was conducted at the First Industrial Park Experimental Station (41°43′40”N, 111°36′6″E, and elevation: 1,417.2 m) of Xinyu Seed Industry Co., Ltd. in Wulanhua Town, Siziwang Banner, Inner Mongolia Autonomous Region. This region features a mid-temperate continental monsoon climate with annual precipitation of 313.8 mm and 3,084–3,286 h of sunshine. The soil is classified as Kastanozem (chestnut soil), with a sandy loam texture, deep profile development, and loose structure. The soil exhibits a pH of 7.8, and an organic matter content of 11.12 g/kg. These soil characteristics make it ideal for potato production. During the 2024 growing season, the average temperature was 14°C and wind speed ranged from 3–4 on the Beaufort scale.

### 2.2 Experimental design

The field experiment adopted a single-ridge, unmulched cultivation system with drip irrigation, using ridges spaced 90 cm apart and plants 16 cm apart within each 6-meter ridge. Each plot contained five ridges, yielding a planting density of 69,444 plants/ha. Four treatments replaced 0% (CK), 40% (T40), 60% (T60), and 80% (T80) of synthetic nitrogen with organic fertilizer (OF; 7.06% N, 1.15% P₂O₅, 1.14% K₂O), as detailed in Table 1. Synthetic fertilizers included urea (46% N), diammonium phosphate (DAP; 18% N, 46% P₂O₅), and potassium sulfate (SOP; 52% K₂O). To maintain constant nutrient inputs (250 kg N, 200 kg P₂O₅, 300 kg K₂O/ha), OF nutrient contributions were calculated first. Phosphorus deficits were initially supplemented with DAP, and remaining nitrogen and potassium with urea and SOP. For T80, DAP was applied only until total nitrogen reached 250 kg/ha, with further phosphorus from monopotassium phosphate (PDP; 52% P₂O₅, 34% K₂O), and remaining potassium from SOP. The potato cultivar V7 was planted mechanically and fertilized manually. The experiment followed a randomized complete block design with three replicates per treatment.

**Table 1:** Gradient design of organic fertilizer application.

Treatment	Urea (kg·ha^−1^)	SOP (kg·ha^−1^)	DAP (kg·ha^−1^)	PDP (kg·ha^−1^)	OF (kg·ha^−1^)	Total fertility (kg·ha^−1^)	$\frac{\text{OF}\times 7.06\%}{250}\times 100\%$
CK	373.35	600.00	434.78	0	0	1408.13	0%
T40	169.81	545.87	399.37	0	1416.43	2531.48	40%
T60	68.04	530.34	381.67	0	2124.65	3104.7	60%
T80	0	464.97	277.78	76.24	2832.86	3651.85	80%

Organic fertilizer (OF): 7.06% N, 1.15% P₂O₅, and 1.14% K₂O. CK: 0% OF, T40: 40% OF, T60: 60% OF, and T80: 80% OF. Synthetic fertilizers: urea (46% N), diammonium phosphate (DAP: 18% N and 46% P₂O₅), potassium sulfate (SOP: 52% K₂O), and monopotassium phosphate (PDP: 52% P₂O₅ and 34%K₂O) used as needed to maintain balanced nutrient inputs.

### 2.3 Rhizosphere soil collection

Rhizosphere soil samples were collected during three critical stages of potato development: seedling (June 24, 2024), tuber initiation (July 15, 2024), and tuber bulking (August 15, 2024). For each of the four treatments, three potato plants exhibiting similar growth vigor were randomly selected and excavated using a spade to approximately 20 cm depth and 5–10 cm distance from the stem to ensure complete root system recovery with adhering soil. After removing loosely attached soil aggregates and excising aboveground plant material, the intact root systems with closely associated rhizosphere soil were immediately preserved on dry ice, yielding 12 total samples across all treatments. In the laboratory, soil adhering to root surfaces was gently removed using a fine brush and collected onto sterile paper, with the three replicate samples from each treatment thoroughly homogenized and subdivided into three equal portions (5–6 g each) before being placed in separate sterile plastic bags with appropriate labeling (Morgan et al., 2018). These samples were then stored at −80°C for the extraction of soil DNA and metabolite analysis (Cindy et al., 2012).

### 2.4 Amplification and sequencing of 16S rDNA

The total DNA was extracted from approximately 0.5 g of rhizosphere soil sample using the E. Z. N. A.® Soil DNA Kit (Omega Biotek, D5625-01). DNA purity and concentration were evaluated using a NanoDrop 2000 spectrophotometer and 2% agarose gel electrophoresis. The V3-V4 regions of bacterial 16S rRNA genes were amplified via PCR using primers 341F (5’-CCTACGGGNGGCW GCAG-3′) and 806R (5’-GGACTACHVGGGTWTCTAA T-3′), while the fungal ITS1 region was amplified with primers ITS1F (5’-CTTGGTCATTTAGAGGAAGTAA-3′) and ITS1R (5’-GCTGCG TTCTTCATCG ATGC-3′). Amplicons (400–450 bp for bacteria, 310 bp for fungi) were purified via agarose gel electrophoresis. Sequencing libraries were prepared using the Illumina TruSeq Nano DNA LT Kit, incorporating dual-index adapters for multiplexed analysis.

### 2.5 Microbial community analysis

Paired-end sequencing (2 × 250 bp) was conducted using the Illumina NovaSeq 6,000 platform, generating raw 16S rRNA and ITS reads for bacterial and fungal communities, respectively. Demultiplexing and merging of the raw reads were performed using FLASH (v1.2.11) with a 10% mismatch tolerance, resulting in consensus sequences of 400–450 bp for bacteria and approximately 310 bp for fungi. Following initial processing, sequences underwent quality filtering with fastp (v0.19.6) and further merging with FLASH (v1.2.7). High-quality sequences were subsequently de-noised using the DADA2 plugin within the QIIME2 (v2021.4) pipeline, which resolves amplicon sequence variants (ASVs) at single-nucleotide resolution based on sample-specific error profiles. Taxonomic classification of bacterial ASVs was performed against the SILVA 16S rRNA database (v138) using the RDP classifier (v2.2), while fungal sequences were annotated using the UNITE v9.0 database.

Microbial diversity analyses were conducted at a uniform sequencing depth. Alpha diversity metrics, including observed ASVs, Chao1 richness, and Shannon index, were calculated using Mothur (v1.30.1), while beta diversity was assessed based on weighted UniFrac distances using Bray-Curtis-based PCoA ordination and PERMANOVA validation. The top 10 most abundant genera were profiled to elucidate treatment-induced shifts in community composition. Multivariate analyses, including ANOVA and PERMANOVA, were employed to evaluate the effects of experimental conditions on dominant phyla and genera. All sequencing and bioinformatic analyses were performed in triplicate to ensure reproducibility.

### 2.6 Yield trait measurements

Potato harvesting was carried out on October 2, 2024, at physiological maturity. From each plot, three uniformly growing potato plants were randomly selected and excavated, yielding a total of nine plants across the three plots. To ensure data integrity, care was taken to minimize tuber damage during excavation, and adhering soil was gently removed from the tubers. For each plant, both the number and total weight of tubers were measured and recorded. The total potato yield was calculated, with total yield expressed as the average tuber weight per plant multiplied by the number of plants in the plot (kg/m^2^).

### 2.7 Statistical analysis

The polynomial regression analysis examining the relationship between potato yield (Y) and organic fertilizer application (X) was conducted using Python, employing the model formulation Y = β_0_ + β_1_ X + β_2_ X^2^ + ℇ. Model parameters (β_0_, β_1_, β_2_) were estimated via ordinary least squares (OLS) using the “numpy.polyfit” function, with model fit assessed through the coefficient of determination (R^2^). The overall statistical significance of the regression model was evaluated using analysis of variance (ANOVA) with F-tests, where *p* < 0.05 served as the threshold for rejecting the null hypothesis. Correlation analyses utilized Pearson’s correlation for normally distributed variables (confirmed by Shapiro–Wilk tests with *p* > 0.05) and Spearman’s rank correlation for non-parametric data, with all correlation matrices subjected to Benjamini-Hochberg false discovery rate (FDR) correction (q < 0.1) to adjust for multiple comparisons. The relationships between microbial community and potato yield were further analyzed through SparCC cooccurrence networks, applying thresholds of∣*ρ*∣ > 0.6 and *p* < 0.01 for network edge inclusion.

## 3 Results

### 3.1 Overall analysis of 16S rDNA and ITS

High-throughput sequencing of bacterial 16S rDNA (V4 region) and fungal ITS1 regions across 12 rhizosphere soil samples generated robust datasets for microbial community analysis. Bacterial sequencing with 41F-806R primers produced 1,470,323 raw reads, yielding 1,216,234 high-quality non-chimeric ASVs after filtering and denoising, with an average of 101,352 ASVs per soil sample (Supplementary Table S1). Fungal sequencing with ITS1F-ITS1R primers generated 1,717,472 raw reads, resulting in 1,617,588 non-chimeric ASVs, averaging 134,799 ASVs per soil sample (Supplementary Table S2). These results provide reliable datasets for downstream diversity and community structure analyses.

### 3.2 Alpha diversity analysis of potato rhizosphere microbial communities

Alpha diversity of potato rhizosphere microbial communities was assessed across seedling, tuber initiation, and tuber expansion stages under different organic fertilizer treatments (Table 2). Good’s Coverage values exceeded 0.995 for bacteria and 0.999 for fungi across all treatments and growth stages, confirming adequate sequencing depth for reliable diversity assessment.

**Table 2:** Alpha diversity analysis of potato inter-root microorganisms under different organic fertilizer treatments across growth periods.

strains	Treatment	Seedling stage				Tuberogenesise				Tuber expansion stage			
		Chao1	Shannon	Ace	Goods-coverage	Chao1	Shannon	Ace	Goods-coverage	Chao	Shannon	Ace	Goods-coverage
Bacteria	CK	5318.74Aab	11.07Aa	5342.96Aab	0.9962Aa	4286.28Aa	10.89Aa	4343.92Aa	0.9955Aa	5537.10Ab	9.91Bb	5581.95Ab	0.9978Aa
	T40	5425.26Aab	11.17Aa	5456.00Aab	0.9963Aa	4295.50Aa	10.75Ab	4348.14Aa	0.9958Aa	7448.55Aa	11.39Aa	7583.13Aa	0.9917Aab
	T60	5605.63Aa	11.21Aa	5657.35Aa	0.9958Aa	4451.29Aa	10.93Aa	4502.48Aa	0.9962Aa	7619.64Aa	11.61Aa	7734.94Aa	0.9939Aab
	T80	4598.57Ab	11.01Aa	4613.63Ab	0.9981Aa	4325.86Aa	10.89Aa	4358.77Aa	0.9970Aa	7982.33Aa	11.69Aa	8145.99Aa	0.9910Ab
Fungi	CK	650.68Aab	4.89Bb	649.81Aab	0.99984Aa	601.92Abc	6.01Bb	603.33Abc	0.9998Aa	356.24Bc	4.36Bb	358.21Bc	0.99991Aa
	T40	552.34Ab	5.64ABb	552.31Ab	0.99995Aa	727.61Aab	7.12Aa	728.25Aab	1.0000Aa	624.86Ab	4.85Bb	625.85Ab	0.99994Aa
	T60	866.03Aa	6.91Aa	866.62Aa	0.99993Aa	789.80Aa	6.94Aa	790.53Aa	0.9998Aa	726.52Aab	6.30Aa	727.42Aab	0.99997Aa
	T80	706.71Aab	6.76Aa	706.84Aab	0.99996Aa	522.90Ac	5.16Cc	523.74Ac	0.9998Aa	793.83Aa	6.69Aa	794.51Aa	0.99997Aa

Unit, piece. Values are the mean of three replicates. CK: 0% OF, T40: 40% OF, T60: 60% OF, and T80: 80% OF. According to Spass of Duncan’s test, the average of the different letters (such as a, b, and c) in each column was significantly different at *p* < 0.05. the average of the different letters (such as A, B, and C) in each column was highly significantly different at *p* < 0.01.

Bacterial richness indices (Chao1 and ACE) varied temporally, peaking during tuber expansion, followed by the seedling stage, with lowest values at tuber initiation. Within each growth period, bacterial richness increased progressively with organic fertilizer application rates (T80 > T60 > T40 > CK). This positive relationship between bacterial richness and fertilizer application remained consistent across all developmental stages. Shannon diversity indices remained stable across treatments and growth stages, indicating that fertilization affected species richness but not overall community evenness.

Fungal communities exhibited distinct patterns compared with bacterial assemblages. During the seedling stage, T60 showed highest fungal richness, followed by T80, while T40 had lower diversity. At tuber initiation, T60 maintained peak fungal richness, followed by T40, while T80 showed the lowest values. By tuber expansion, T80 exhibited substantially increased fungal richness, reaching the highest observed values. T60 maintained consistently high fungal richness throughout all developmental stages.

Furthermore, Fungal Shannon indices were consistently lower than bacterial indices across all growth stages, approximating half the bacterial community values. CK and T40 maintained lower diversity indices, while T60 and T80 showed significantly higher values (T80 > T60 > T40 > CK).

### 3.3 Beta diversity of microbial Community in Rhizosphere Soils

**Figure 1:**
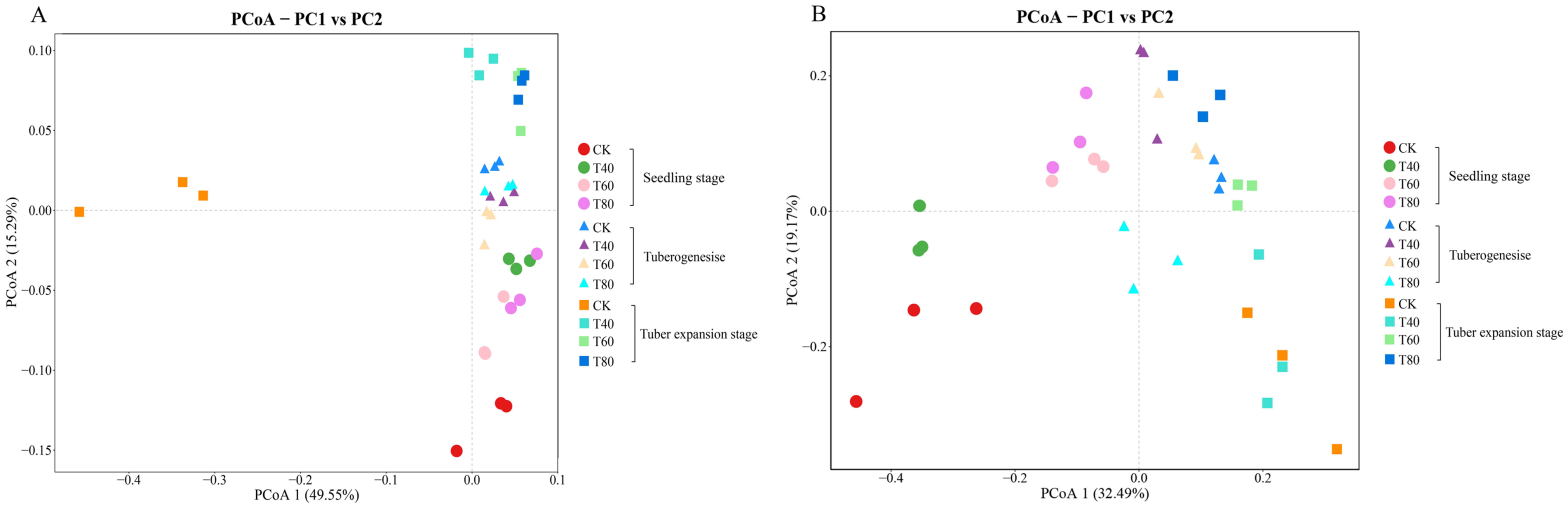
Principal coordinates analysis (PCoA) of rhizosphere microbial communities. PCoA plots based on Bray-Curtis distances showing (**A**) bacterial communities and (**B**) fungal communities at the genus level in potato rhizosphere. Colors represent organic fertilizer treatments. Symbols indicate growth stages: circles = seedling, triangles = tuberogenesis, squares = tuber expansion.

Principal Coordinate Analysis (PCoA) based on ASV composition at the genus level revealed distinct compositional patterns in bacterial and fungal communities across organic fertilization treatments and developmental stages. Sample clusters separated according to fertilization regimes, demonstrating pronounced effects of organic fertilizer application on microbial community structure. For bacterial communities, PCoA1 and PCoA2 explained 49.55 and 15.29% of total variance, respectively (Figure 1A). Fungal community ordination showed PCoA1 and PCoA2 explaining 32.49 and 19.17% of variance, respectively (Figure 1B). These high variance explanations indicate that the ordination effectively captures microbial community differentiation patterns.

Microbial communities exhibited stage-specific temporal dynamics in response to organic fertilization. During the seedling stage, bacterial and fungal communities showed pronounced dispersion across fertilization treatments, indicating differential establishment of rhizosphere assemblages. Bacterial communities progressively converged through developmental stages, clustering cohesively during tuber expansion in the upper ordination space (Figure 1A). The control treatment (CK) maintained distinctly segregated positions throughout all stages, particularly in bacterial communities where CK samples consistently occupied the negative PCoA1 region. Fungal communities exhibited greater heterogeneity than bacterial assemblages, especially during tuberogenesis, suggesting taxon-specific sensitivities to organic inputs (Figure 1B).

### 3.4 Alterations in bacterial and fungal community composition

**Figure 2:**
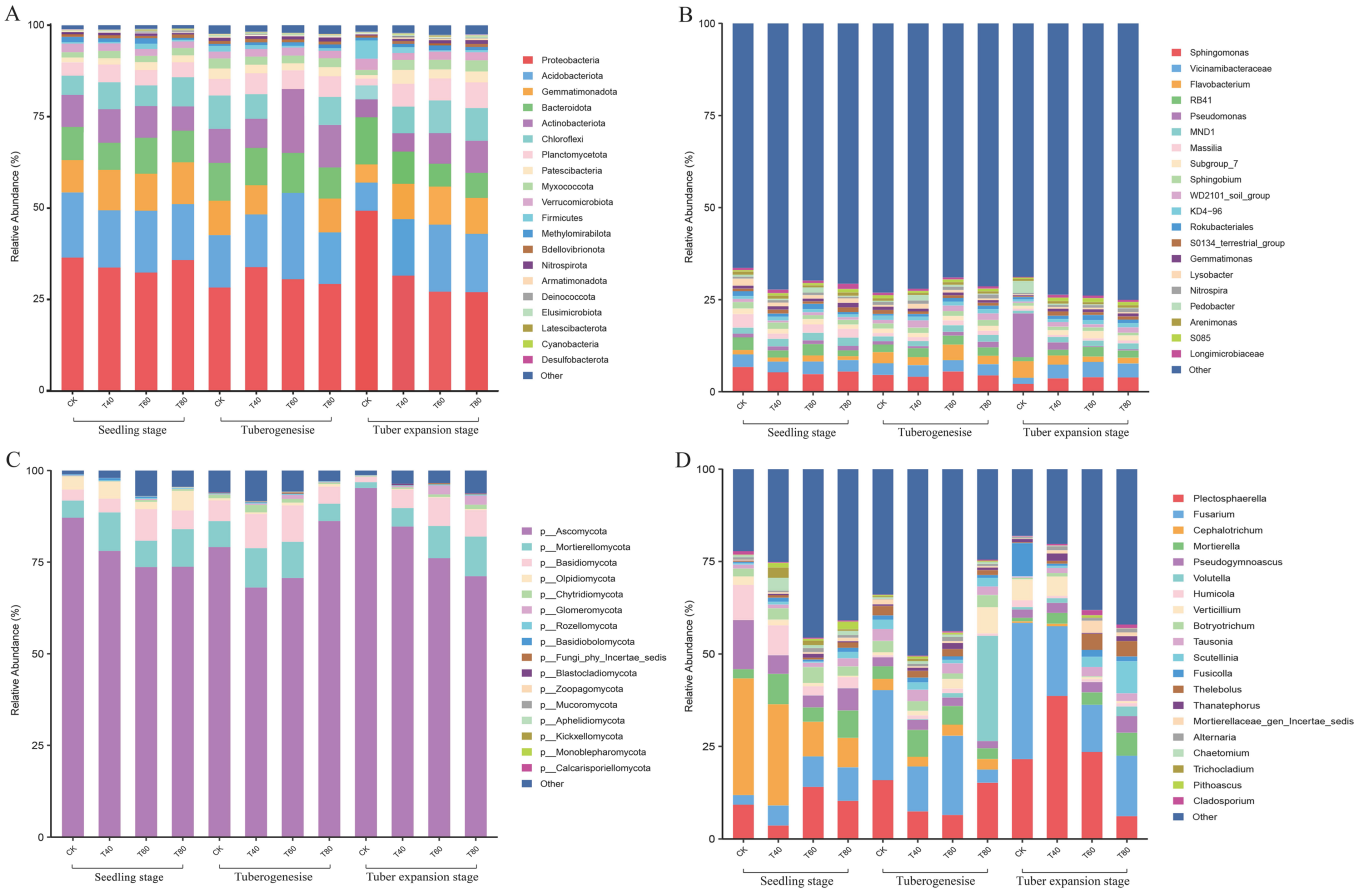
Rhizosphere microbial community composition of potatoes at different growth stages under varying organic fertilizer treatments. Relative abundance (%) of bacterial communities at **(A)** phylum and **(B)** genus levels, and fungal communities at **(C)** phylum and **(D)** genus levels in potato rhizosphere soil. Communities were analyzed across three developmental phases (seedling, tuberogenesis, and tuber expansion stages) and four organic fertilizer treatments (CK, T40, T60, and T80). Bar charts display the top 20 most abundant taxa. The y-axis shows relative abundance percentages.

The taxonomic composition of bacterial and fungal communities in the potato rhizosphere exhibited distinct temporal dynamics across different growth stages and Organic fertilizer treatments. Bacterial communities were dominated by four phyla: Proteobacteria, Acidobacteriota, Gemmatimonadota, and Bacteroidota. Proteobacteria dominated both CK and T80 during the seedling stage but declined progressively through tuber formation and expansion stages. Conversely, Planctomycetota increased markedly during tuber expansion (Figure 2A). At the genus level, *Sphingomonas*, *Vicinamibacteraceae*, *Flavobacterium*, and *RB41* were the most abundant genera. The relative abundances of *Sphingomonas*, *Pseudomonas*, and *Massilia* decreased during potato development, while *Vicinamibacteraceae* increased, particularly under T60 and T80 during the tuber expansion. *Flavobacterium* and *RB41* remained stable across developmental stages and treatments (Figure 2B).

Fungal communities consisted primarily of *Ascomycota*, Mortierellomycota, Basidiomycota, and Olpidimycota, collectively accounting for ~95% of total abundance. *Ascomycota* alone represented 75–90% of fungal communities. During potato development, Olpidimycota decreased sharply while *Ascomycota* and Basidiomycota increased gradually. Glomeromycota emerged prominently in T60 and T80 during tuber expansion. *Ascomycota* abundance followed the pattern CK > T40 > T60 > T80 at both seedling and expansion stages, while Mortierellomycota remained unchanged (Figure 2C). At the genus level, *Plectosphaerella*, *Fusarium*, *Cephalotrichum*, and *Mortierella* dominated. Increasing organic fertilizer application correlated with declining *Cephalotrichum* and increasing *Fusarium* and *Plectosphaerella* during the seedling stage. This trend continued through tuber formation, with *Cephalotrichum* declining and *Fusarium* increasing. During tuber expansion, both *Plectosphaerella* and *Fusarium* increased further, ranking T40 > T60 > CK > T80. The T80 consistently enriched *Scutellinia* throughout the growth period (Figure 2D).

### 3.5 LEfSe analysis of bacterial and fungal communities

**Figure 3:**
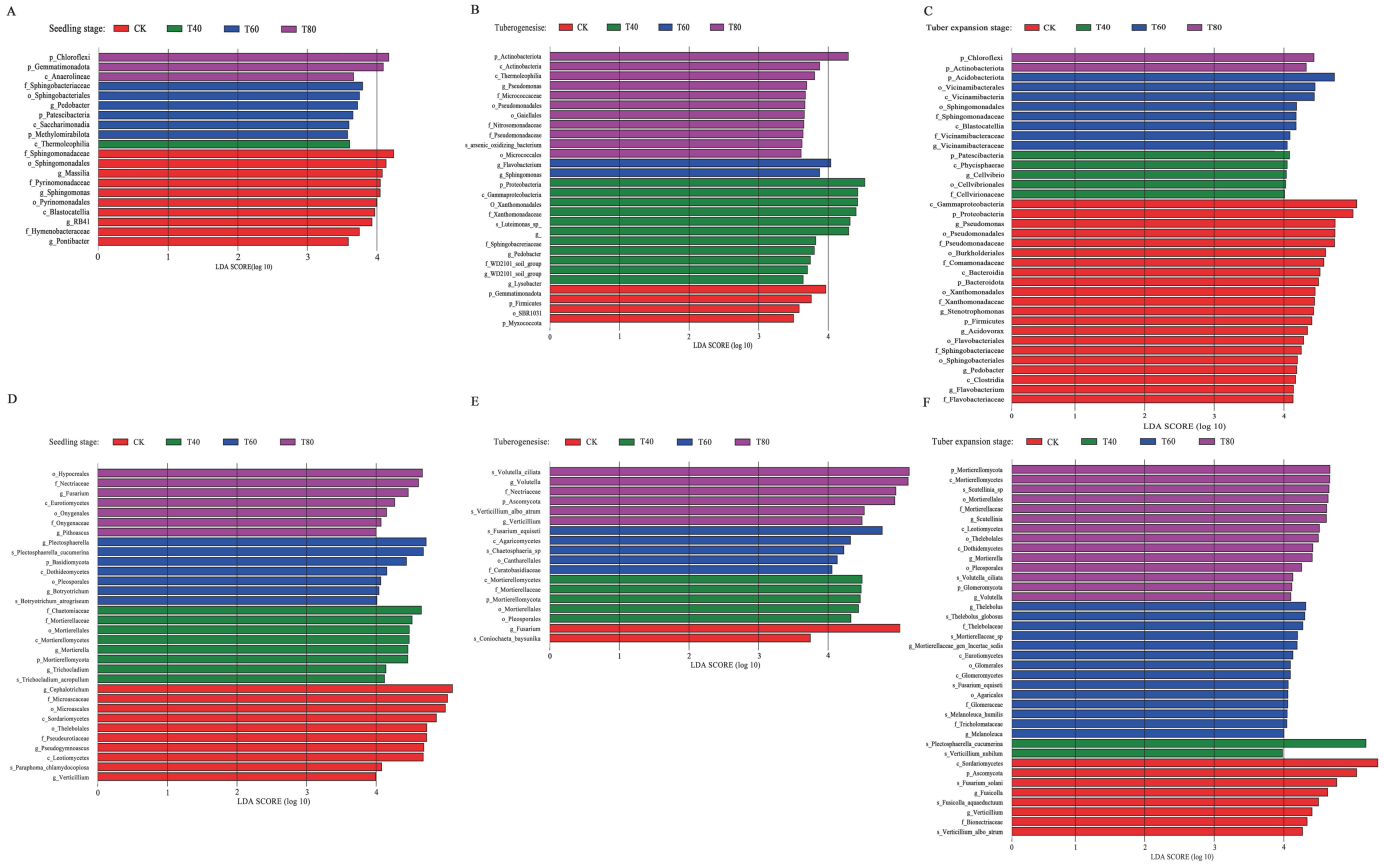
Linear discriminant analysis effect size (LEfSe) of microbial communities in potato rhizosphere soil under different organic treatments (CK, T40, T60, and T80) across three growth stages. **(A–C)** show differential abundance of rhizobacterial communities (LDA score >3.5) during seedling stage **(A)**, tuberogenesis **(B)**, and tuber expansion stage **(C)**. **(D–F)** display differential abundance of rhizofungal communities during seedling stage **(D)**, tuberogenesis **(E)**, and tuber expansion stage **(F)**. Bar colors represent the specific organic treatment where each taxon was significantly enriched, while bar length indicates the magnitude of differential abundance between treatments.

LEfSe analysis revealed stage-specific shifts in potato rhizosphere microbial communities under varying organic fertilization regimes. During the seedling stage, organic treatments (T40, T60, and T80) showed reduced bacterial enrichment compared with the control (CK). The CK was dominated by f_Sphingomonadaceae, o_Sphingomonadales, and g_Massilia. Organic amendments preferentially selected for p_Chloroflexi and p_Gemmatimonadota phyla, likely due to fertilizer-induced increases in soil polysaccharides. These conditions favored cellulose-degrading genera such as g_*Levilinea* and g_*Roseiflexus* (Figure 3A). At tuber formation, T60 showed limited bacterial enrichment, with only g_*Flavobacterium* and g_*Sphingomonas* reaching significance (LDA > 3.5). In contrast, T80 promoted *p_*Actinobacteriota and f_Pseudomonadaceae lineages. These included nitrogen-cycling o_Micrococcaceae and antibiotic-producing g_*Pseudomonas*, which aligned with increased nitrogen demand and improved soil structure (Figure 3B). During tuber expansion, T60 enriched *p_*Acidobacteriota, o_Vicinamibacterales, and f_Sphingomonadaceae. Meanwhile, T80 selection pressure favored specialized p_*Chloroflexi* taxa adapted to high organic carbon environments (Figure 3C).

Fungal communities displayed treatment-dependent functional succession throughout potato development. Each organic amendment rate shaped distinct microbial assemblages at different growth stages. At the seedling stage, T40 enhanced plant growth-promoting fungi, including Mortierella and Mortierellomycetes (Figure 3D). During tuber formation, T60 favored ecological specialists such as Fusarium equiseti and Ascomycota. T80 led to enrichment of saprophytic and weakly pathogenic taxa, including Volutella and Nectriaceae (Figure 3E). By the expansion phase, T60 promoted phosphorus acquisition through Glomeromycota mycorrhizae and maintained Mortierella-driven decomposition of organic intermediates (Figure 3F). Collectively, these results demonstrate that T60 achieves an optimal balance of microbial community structure and function, fostering beneficial fungi that support nutrient cycling and potato growth across developmental stages.

### 3.6 Potato yield under different potassium treatments

**Figure 4:**
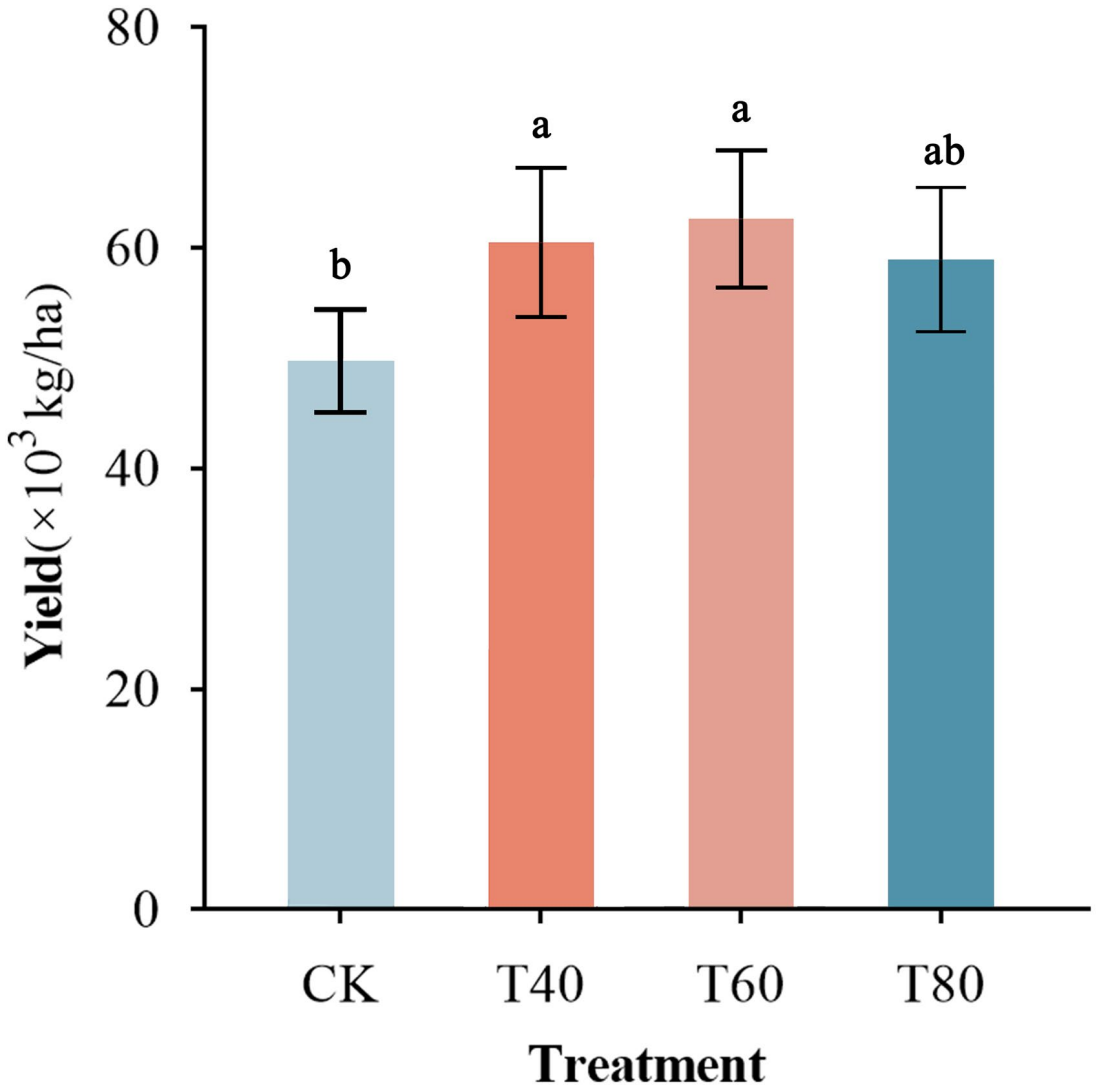
Potato yield in response to different organic fertilizer application rates. Bars with different letters (a,b) indicate statistically significant differences (*p* < 0.05). Error bars represent standard deviation.

Moderate organic fertilizer application significantly enhanced potato yield, exhibiting a quadratic response whereby yield increased with organic fertilizer substitution up to 60% and then declined at higher rates. Specifically, T60 produced the highest yield (64,886 kg/ha), which was markedly greater than the control (49,824 kg/ha), while T40 and T80 yielded 60,507 kg/ha and 58,901 kg/ha, respectively. Although these three treatments did not differ significantly from one another, all outperformed the control (Figure 4).

### 3.7 Correlation analysis of potato yield and microbial diversity

**Figure 5:**
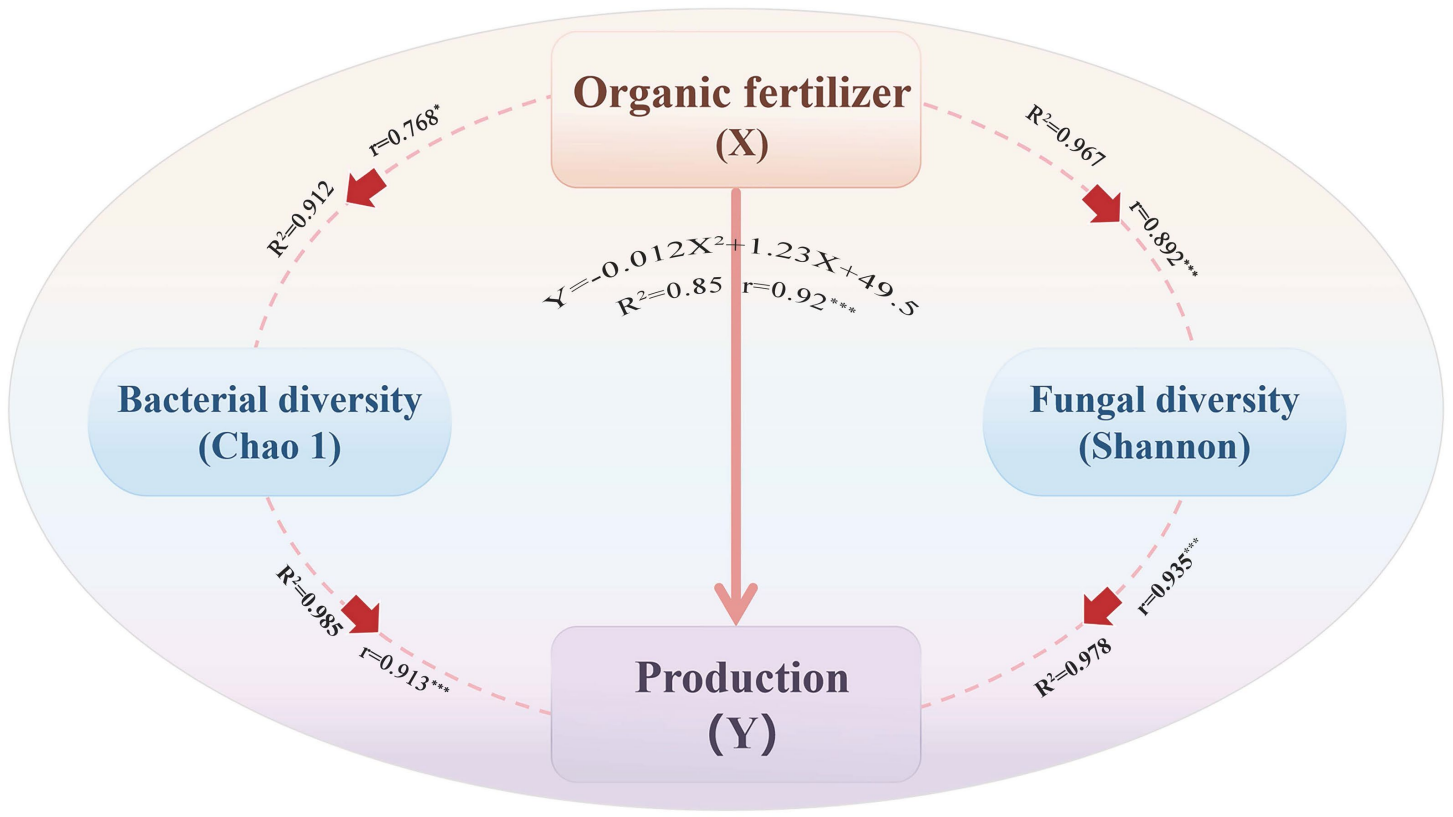
Structural equation model showing relationships between potato yield, soil microbial diversity, and organic fertilizer application. *p < 0.5, ***p < 0.01.

Regression analysis revealed a quadratic relationship between potato yield and organic fertilizer application using Python’s statsmodels package. Yield increased with fertilizer rate until reaching an optimal point, then declined at higher rates. The model predicted maximum yield (81,020 kg/ha) at 51.25% organic fertilizer substitution (Figure 5). Beyond this threshold, yields declined progressively with further increases in fertilizer application, following a quadratic pattern where greater deviations from the optimum resulted in steeper yield reductions.

Microbial diversity exhibited similar patterns. Bacterial richness (Chao1) peaked at approximately 60% fertilization. Bacterial diversity correlated strongly with yield, likely through enhanced enzyme activity that improves nutrient cycling. Fungal diversity (Shannon) also demonstrated a quadratic relationship with yield, with optimal yield occurring at Shannon index values of 6.60. This suggests a balance between beneficial fungal symbiosis and risks from competition or disease at higher fungal diversity levels. The optimal fertilizer application rate for both yield and microbial diversity ranged between 55–60%. At this level, bacterial nitrifiers provided most plant-available nitrogen while fungal hyphae delivered acquired phosphorus to roots.

## 4 Discussion

This study examined potato rhizosphere microbial communities across growth stages using organic fertilizer rates (0, 40, 60, 80% synthetic nitrogen replacement). PCoA analysis revealed distinct separations between organic treatments and controls, maximum differentiation during tuber expansion under 80% treatment, indicating that higher organic fertilizer doses induce more pronounced community changes. Bacterial Chao1 indices peaked at 60% during tuber formation and rose progressively with rates during expansion. Fungal Shannon indices maximized at 60% during seedling and tuber formation stages. These align with studies showing moderate organic substitution boosts microbial abundance and diversity (Quan et al., 2023). The mechanism involves balanced C: N ratios from appropriate fertilizer, while excess causes rapid nitrogen mineralization and carbon limitation, peaking abundance at 60% before declining at 80% (Lei et al., 2022).

Bacterial communities were dominated by Proteobacteria, Actinobacteriota, and Patescibacteria, with PGPR genera *Pseudomonas* and *Sphingomonas* increasing under 60 and 80% treatments via auxin secretion, siderophore production, and pathogen control (Das et al., 2022). Fungal communities featured Mortierellomycota and Ascomycota, adept at decomposition and growth promotion (Rubee et al., 2020). At 60%, disease-resistant *Plectosphaerella* rose while harmful *Cephalotrichum* fell, showing synergy with plant defenses (Ning et al., 2022). LEfSe analysis revealed stage-specific changes, with organic treatments favoring Chloroflexi and Gemmatimonadota during the seedling stage due to fertilization-induced increases in soil polysaccharides that support cellulose-degrading genera (Bose et al., 2022). During tuber formation, 60% favored fungi in high-carbon conditions, while 80% boosted nitrogen-cycling and antibiotic-producing bacteria. This suggests that high carbon loading created conditions favoring fungi while suppressing most bacterial taxa (Quan et al., 2023). Moderate rates (40–60%) thus optimize structure by promoting beneficial microbes and suppressing pathogens (Wenyi et al., 2023).

Organic fertilizer rates positively affected microbial diversity and yield, with fertilizer proportion correlating strongly (*r* = 0.92, R^2^ = 0.85). Bacterial Chao1 and fungal Shannon indices correlated with inputs, enhancing richness via better nutrients (Quan et al., 2023). The 60 and 80% treatments enriched decomposition-related Actinobacteria and Ascomycota during expansion. Diversity-yield correlations were strong (bacterial Chao1: *r* = 0.913; fungal Shannon: *r* = 0.935), confirming diversity drives yield (Martins et al., 2024). Mechanisms include enriched phosphorus-solubilizing and nitrogen-fixing groups (Shaojing et al., 2022), with *Vicinamibacteraceae* and *Nitrosomonadaceae* providing nutrients (Chengyu et al., 2023).

Regression showed quadratic yield response, peaking at 81,020 kg/ha with 51.25% fertilizer. Beyond this, 10% increases caused quadratic declines, steeper with greater excess. Optimal rates for yield and diversity were 55–60%, where nitrifiers supply nitrogen and fungal hyphae deliver phosphorus. Thus, 40–60% substitution optimizes yield via enhanced diversity, supporting precision application (Sisay and Sisay, 2019). Moderate organic fertilization supports complementary microbial functions while over-application disrupts essential microbial networks needed for sustainable yields.

## Data Availability

The original contributions presented in the study are publicly available. This data can be found at: https://www.ncbi.nlm.nih.gov/sra, accession number SRP579091.
