# Supplementary material for: Organic fertilizer application rates affect rhizosphere microbial communities and yield optimization in potato (Solanum tuberosum L. V7)
Source: Front Microbiol. 2025 Aug 7;16:1651178. doi: 10.3389/fmicb.2025.1651178 (PMC12372700; doi:10.3389/fmicb.2025.1651178)
Supplement: Supplementary file 1 [file Table_1.docx]

**Table S1** Bacterial statistical data

| Periods | Treatment | input | filtered | denoised | non-chimeric |
| --- | --- | --- | --- | --- | --- |
| Seedling stag | CK | 163512 | 155461 | 145915 | 145598 |
|  | T40 | 155969 | 148527 | 138356 | 138048 |
|  | T60 | 154270 | 146647 | 134659 | 134061 |
|  | T80 | 135002 | 127885 | 119965 | 119789 |
| Tuberogenesis | CK | 86568 | 79936 | 67513 | 67173 |
|  | T40 | 83180 | 77271 | 65362 | 64914 |
|  | T60 | 82731 | 76772 | 63294 | 62936 |
|  | T80 | 81665 | 75795 | 62945 | 62616 |
| Tuber expansion stage | CK | 119165 | 113552 | 97635 | 91782 |
|  | T40 | 141392 | 136099 | 117558 | 115851 |
|  | T60 | 126070 | 121334 | 101291 | 99972 |
|  | T80 | 140799 | 135585 | 114805 | 113494 |
| Sum | | 1470323 | 1394864 | 1229298 | 1216234 |

**Table S2** Fungi statistics data

| Periods | Treatment | input | filtered | denoised | non-chimeric |
| --- | --- | --- | --- | --- | --- |
| Seedling stage | CK | 215161 | 208342 | 207857 | 207802 |
|  | T40 | 175660 | 167364 | 166941 | 166917 |
|  | T60 | 168863 | 159079 | 158501 | 158278 |
|  | T80 | 155191 | 148333 | 147893 | 147874 |
| Tuberogenesis | CK | 106874 | 100319 | 99871 | 99424 |
|  | T40 | 92281 | 87311 | 86187 | 86073 |
|  | T60 | 107215 | 99743 | 98882 | 98736 |
|  | T80 | 107261 | 99426 | 98788 | 98446 |
| Tuber expansion stage | CK | 160830 | 155358 | 154673 | 146492 |
|  | T40 | 147080 | 142812 | 142105 | 140176 |
|  | T60 | 143434 | 137825 | 137157 | 136417 |
|  | T80 | 137622 | 131994 | 131190 | 130953 |
| Sum | | 1717472 | 1637906 | 1630045 | 1617588 |
